# A-Mode Ultrasound Bladder Volume Estimation Algorithm Based on Wavelet Energy Ratio Adaptive Denoising

**DOI:** 10.3390/s24061984

**Published:** 2024-03-20

**Authors:** Rencheng Jin, Qipeng Huang, Jiaxu Jiang, Peihao Hu

**Affiliations:** Key Laboratory for Micro/Nano Technology and System of Liaoning Province, Dalian University of Technology, Dalian 116024, China; 18827603081@mail.dlut.edu.cn (Q.H.); jiangjx@mail.dlut.edu.cn (J.J.); 22204093@mail.dlut.edu.cn (P.H.)

**Keywords:** ultrasound, bladder volume, wavelet denoising, cross-correlation analysis

## Abstract

Assessing bladder function is pivotal in urological health, with bladder volume a critical indicator. Traditional devices, hindered by high costs and cumbersome sizes, are being increasingly supplemented by portable alternatives; however, these alternatives often fall short in measurement accuracy. Addressing this gap, this study introduces a novel A-mode ultrasound-based bladder volume estimation algorithm optimized for portable devices, combining efficient, precise volume estimation with enhanced usability. Through the innovative application of a wavelet energy ratio adaptive denoising method, the algorithm significantly improves the signal-to-noise ratio, preserving critical signal details amidst device and environmental noise. Ultrasonic echoes were employed to acquire positional information on the anterior and posterior walls of the bladder at several points, with an ellipsoid fitted to these points using the least squares method for bladder volume estimation. Ultimately, a simulation experiment was conducted on an underwater porcine bladder. The experimental results indicate that the bladder volume estimation error of the algorithm is approximately 8.3%. This study offers a viable solution to enhance the accuracy and usability of portable devices for urological health monitoring, demonstrating significant potential for clinical application.

## 1. Introduction

Due to the continuous advancements in medical technology and the escalating severity of an aging population, monitoring the health status of the urinary system has grown in importance. This holds particularly true for the assessment of bladder function. Urinary incontinence and voiding dysfunction increasingly impair patients’ quality of life [1]. This imposes not only a psychological and economic burden on patients and their families but also poses a significant challenge to the healthcare system.

Bladder urine volume assessment is divided into invasive and non-invasive techniques. The invasive technique involves inserting a catheter into the urethra to allow for urine evacuation and the accurate measurement of volume; however, this method presents significant disadvantages, such as pain, restricted movement, and the risk of secondary urinary tract infections [2,3,4], making its long-term application impractical.

Given the limitations of invasive techniques, researchers globally have increasingly turned to exploring and implementing non-invasive technologies. The principle of non-invasive estimation relies on utilizing sensor waveform signals or generated images, which are computationally processed to measure the bladder urine volume without inflicting harm on the patient’s body [5]. This method boasts advantages such as safety and rapidity; however, the majority of non-invasive instruments currently utilized in clinical diagnosis are bulky and not wearable.

In the contemporary era, with the ongoing advancement of modern medical technologies, portable bladder urine assessment technology has witnessed significant and rapid development. The principal sensing methodologies in portable devices include ultrasound, near-infrared spectroscopy [6,7], and bioimpedance [8,9]. Among these technologies, ultrasound sensing is the predominant method. Numerous scholars, both domestically and internationally, have achieved significant research advancements. Petrican et al. introduced a miniature ultrasound bladder monitor that captures the echo signal through hardware and employs echo time as a warning threshold through a time window function. This accuracy of the system is approximately 70% [10]. A study by Kristiansen et al. involved experimental measurements of the bladder urine volume and the span between the anterior and posterior walls of the bladder in a varied cohort. A subsequent analysis of the measured volume using a regression analysis enhanced the estimation accuracy of the bladder urine volume; however, the measurements exhibited lower accuracy in female subjects [11]. Tanaka et al. divided the anatomical model of the bladder into three distinct sections: a cone, a cylindrical segment, and a hemisphere. Multiple ultrasound transducers were employed to ascertain the bladder wall distance by capturing the echo signals. Significant changes in the shape of the bladder occurred during filling [12]. Haijun Niu et al. utilized an ultrasound probe to measure the depth and height of the bladder, estimating the urine volume within the bladder using the proposed algorithm [13]. Simultaneously, Van Leuteren et al. presented the URIKA Bladder Monitoring (UBM) system, designed to measure the bladder length of a child using a pediatric wearable ultrasound monitor [14]. Commercially available wearable ultrasound devices aid in managing the voiding cycle of a patient; however, these devices are incapable of estimating the bladder volume [15].

In the existing research, although ultrasonic techniques have yielded significant achievements in various fields, the development of ultrasonic-based portable technology for estimating bladder urine volume predominantly remains in the theoretical research phase. Furthermore, there exists a notable disparity in measurement accuracy when compared with clinical-grade devices.

In this study, we introduce a bladder volume estimation algorithm for A-mode ultrasound-based portable devices to address the issue of suboptimal measurement accuracy, enhancing the bladder volume measurement precision. Acknowledging the attenuation and scattering of the echo signals in the abdomen, which introduces additional noise, an enhanced wavelet adaptive threshold function is proposed for signal denoising. Utilizing the Gauss–Newton algorithm, a reference signal was constructed to obtain the cross-correlation function through an operation on the denoised echo signal. During this process, the energy and amplitude characteristics of the echo signal facilitate the estimation of the reference signal parameters, accelerating their convergence. Ultimately, ellipsoid fitting utilizing the position information from the anterior and posterior walls of the bladder calculates the ellipsoid volume. Experimental validation with a porcine bladder demonstrated significant improvements in the bladder volume estimation accuracy. 

This paper is organized as follows: Section 2 elucidates the principles of ultrasonic measurement of the bladder volume and wavelet threshold transformation. Section 3 provides an exhaustive description of the bladder volume estimation algorithm, divided into three main components: signal denoising, feature extraction, and volume fitting. Section 4 details the experimental design and validation using a porcine bladder, demonstrating that the algorithm significantly enhances the accuracy of the bladder volume estimation. Section 5 presents the conclusion.

## 2. Theoretical Basis

### 2.1. Principle of Ultrasound Bladder Volume Estimation

**Figure 1:**
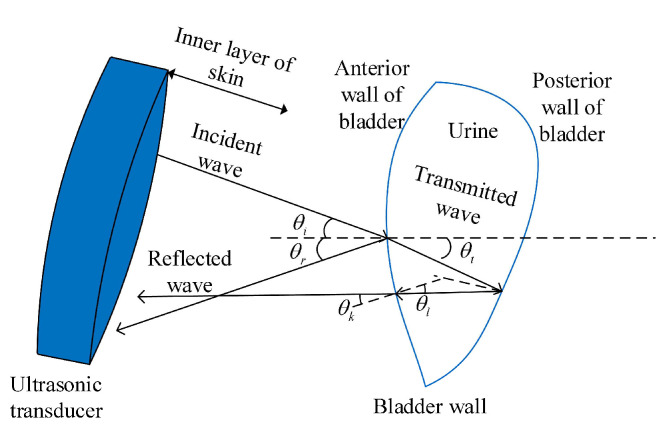
Interaction of the ultrasound and the bladder.

Ultrasound technology quantifies the bladder urine volume through the reflection of sound waves, utilizing their echogenic properties for the measurement. An ultrasonic transducer generates and receives ultrasonic signals, converting the electrical energy into ultrasonic waves and back into electrical signals as echoes. As the ultrasonic waves traverse the human tissues, they generate reflected waves upon interacting with various tissue structures. The bladder, a fluid-filled cavity, reflects the ultrasound waves at the interface between its wall and the contained fluid when it is filled with urine. By analyzing the echo intensity produced by the fluid inside the bladder and its reflection characteristics, it is possible to gauge the volume of the bladder. Figure 1 illustrates the interaction process between the ultrasound waves and the bladder.

**Figure 2:**
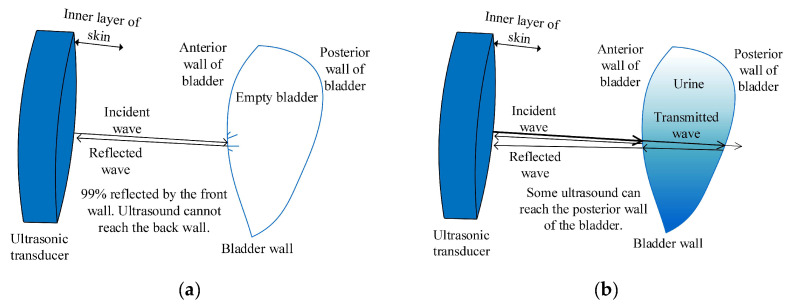
(**a**) Interaction of the ultrasound with an empty bladder; (**b**) Interaction of the ultrasound with a bladder filled with urine.

The ultrasound echo signals exhibit dependency on the physiological state of the bladder, manifesting unique signatures in scenarios where the bladder is vacant compared with when it is replete. Figure 2 delineates the differential interactions between the ultrasound beams and the bladder across these contrasting conditions.

When the bladder is empty, more than 99.9% of the ultrasound pulses are reflected off the anterior wall of the bladder, making it nearly impossible to obtain information about the posterior wall at this time. This high reflection rate is due to the lack of a medium (urine) inside the bladder that would allow the ultrasound waves to pass through and reflect off the posterior wall.

In contrast, when the bladder contains urine, approximately 5% of the ultrasound pulses can penetrate the anterior wall and reach the posterior wall. This penetration is possible because the urine acts as a medium that allows the ultrasound waves to travel through it and reflect off the posterior wall, providing valuable information on the fullness of the bladder and the relative distance between the anterior and posterior walls.

When the ultrasonic waves are emitted by a transducer, the differential in the propagation distance results in the initial reception of the echo signals from the anterior wall of the bladder, succeeded by the echoes from the posterior wall. This phenomenon is represented in the signal by two pronounced peaks. By analyzing the time difference between these peaks, the Time of Flight (ToF) of the ultrasound can be calculated. Subsequently, leveraging the established speed of the ultrasound propagation, the distance separating the anterior and posterior walls of the bladder can be accurately determined. By measuring the anterior and posterior walls of the bladder at different locations, we can obtain multiple sets of data for the anterior and posterior walls of the bladder. Using a suitable algorithm, we can then estimate the volume of the bladder. This method allows for a non-invasive, accurate assessment of the bladder volume, which is crucial for diagnosing and managing urinary tract and bladder conditions.

### 2.2. Principle of Wavelet Threshold Denoising

**Figure 3:**
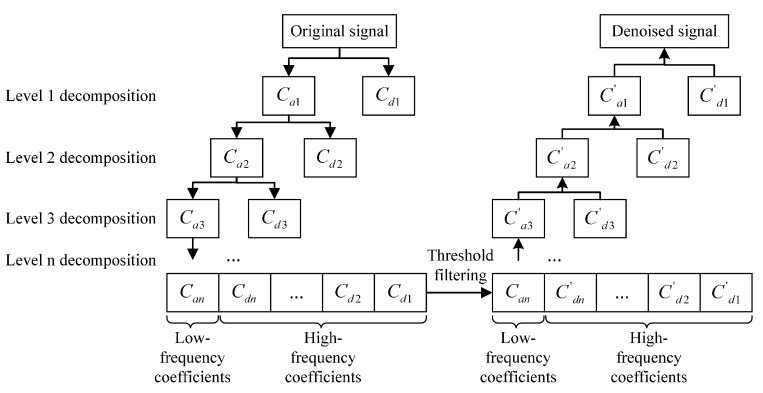
Wavelet thresholding denoising flowchart.

The wavelet thresholding denoising principle leverages the multi-scale decomposition ability of wavelet transforms to separate noise from useful signal information. Initially, a wavelet transform is applied to the signal, breaking it down into wavelet coefficients across various scales. These coefficients at each scale capture the distinct characteristics of the signal at that specific scale. Following decomposition, denoising is accomplished using a thresholding technique applied to these wavelet coefficients, with the threshold determined by the noise profile within the signal. The coefficients falling below this threshold are predominantly noise and are thus zeroed out or diminished, whereas the coefficients above the threshold are preserved, embodying the core signal data. Figure 3 demonstrates the fundamental process of wavelet denoising [16]. The original signal undergoes a level-by-level decomposition into high-frequency coefficients ($C_{d}$) and low-frequency coefficients ($C_{a}$). In the context of the ultrasonic signals, noise predominantly resides within the high-frequency coefficients. The process of denoising these signals effectively involves the application of a threshold function to sift through and selectively filter out the high-frequency coefficients. 

The computation of the wavelet coefficients requires determination using thresholding methods [17]. To ascertain the denoising threshold, this study incorporates the concept of reference noise [18]. A period of equal length was recorded without the emitting ultrasound, utilizing a reference noise signal. Given that Gaussian white noise adheres to a Gaussian distribution, the amplitude is highly likely to fall within the $3\sigma_{j}$ range, in accordance with this distribution law. Consequently, it can be inferred that the signal segment exceeding the $3\sigma_{j}$ amplitude harbors the useful high-frequency components of the noisy signal. This criterion serves to determine the denoising threshold for the signal containing noise at each decomposition level. The value of $\sigma_{j}$ is ascertained by applying Bessel’s formula.

(1) $$\sigma_{j}=\sqrt{\frac{\sum {\left(d_{j}-\bar{d_{j}}\right)}^{2}}{n-1}}$$

where $d_{j}$ is the wavelet coefficient of the j level, and n is the number of wavelet coefficients in the level. The detailed wavelet denoising method is explained in Section 3.1.

## 3. Wavelet Adaptive Denoising Algorithm for Bladder Volume Estimation Based on Energy Ratio

**Figure 4:**
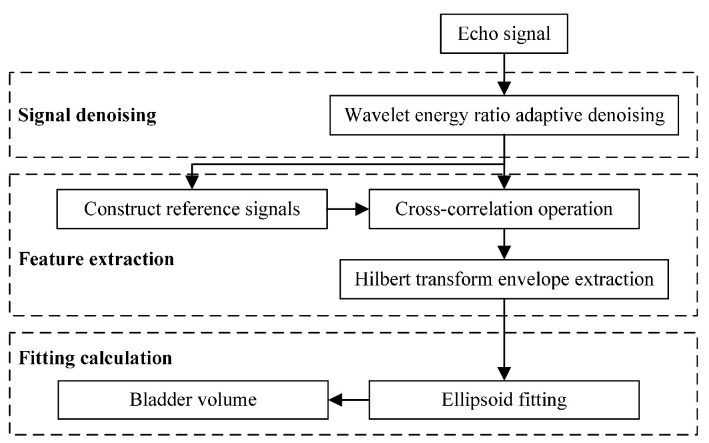
The basic framework of the algorithm.

Figure 4 illustrates the core algorithm for bladder volume estimation described in this study. Initially, to enhance the signal-to-noise ratio (SNR), the non-smooth echo signal is denoised by employing a wavelet-based method predicated on the energy ratio. A Gaussian empirical model is employed to generate a reference signal for the cross-correlation algorithm, utilizing the appropriate initial values. This approach significantly enhances the fit accuracy and reduces the iteration count across diverse SNR settings. The Hilbert transform is subsequently employed to envelope the cross-correlation function and accurately determine the arrival time of the echo. Based on the position data of the anterior and posterior walls of the bladder, an ellipsoid fitting was performed using the least squares method. The volume of the fitted ellipsoid was subsequently calculated to estimate the volume of the bladder.

### 3.1. Wavelet Adaptive Threshold Denoising Algorithm Based on Energy Ratio

#### 3.1.1. Energy Ratio Adaptive Threshold Function

In ultrasound bladder volume monitoring, noise challenges stem mainly from ambient conditions and system limitations. Ambient noise, influenced by human activity, mechanical vibrations, and external disturbances, exhibits a random nature and a broad frequency spectrum, complicating signal interpretation. Systematic noise includes quantization errors during signal digitization, power supply fluctuations affecting the signal integrity, and inherent electronic noise from components, all of which degrade the quality of the ultrasound signals. Addressing these noise sources is crucial for enhancing the accuracy and reliability of the bladder volume assessments.

In this study, we employ the wavelet decomposition technique for the denoising of the echo signals, aiming to attain a high level of precision in noise reduction.

Conventional approaches commonly employ hard and soft thresholding techniques. Both techniques apply a threshold function to process the wavelet coefficients [19]. The following equations show the hard and soft threshold functions, respectively. The hard threshold function expression is given below:

(2) $$d\left(j,k\right)=\left\{\begin{array}{cc} 0, & |d_{j,k}|<\lambda_{j}\\ d_{j,k}, & |d_{j,k}|\geq \lambda_{j} \end{array}\right.$$

The soft threshold function is given in the following equation:

(3) $$d\left(j,k\right)=\left\{\begin{array}{cc} 0, & |d_{j,k}|<\lambda_{j}\\ sgn\left(d_{j,k}\right)\left(\left|d_{j,k}\right|-\lambda_{j}\right), & |d_{j,k}|\geq \lambda_{j} \end{array}\right.$$

where $d_{j,k}$ is the wavelet coefficient of the j level, and k denotes the *k*th decomposition coefficient at that particular level. $\lambda_{j}$ is the threshold of the j level under the wavelet decomposition.

The conventional method involves filtering the coefficients obtained from the wavelet decomposition using soft and hard thresholding functions. This process aims to eliminate the coefficients associated with noise, thereby refining the signal; however, each technique exhibits distinct drawbacks. The hard thresholding technique introduces the unwanted high-frequency noise into the signal, potentially causing oscillations [20]. Conversely, the soft thresholding technique generates a fixed bias in the denoised signal [21].

To overcome the limitations of soft and hard thresholding, numerous threshold functions have been proposed by researchers [22,23,24]. Following an extensive review of the existing literature and empirical validation, this study proposes a variable threshold function. This function dynamically adjusts the hardness or softness according to the energy ratio between the signal and the white noise at a particular decomposition level. The mathematical expression is as follows:

(4) $$d\left(j,k\right)=\left\{\begin{array}{cc} sgn\left(d_{j,k}\right)\frac{{\left|d_{j,k}\right|}^{\beta }}{\beta {\lambda_{j}}^{\beta -1}}+sgn\left(d_{j,k}\right)\frac{2(\beta -1)\lambda_{j}}{\pi \beta }arctan\frac{{\left|d_{j,k}\right|}^{m_{j}}}{{\lambda_{j}}^{m_{j}}}, & |d_{j,k}|<\lambda_{j}\\ d_{j,k}-sgn\left(d_{j,k}\right)\frac{2(\beta -1)\lambda_{j}}{\pi \beta }arctan\frac{{\lambda_{j}}^{m_{j}}}{{\left|d_{j,k}\right|}^{m_{j}}}, & |d_{j,k}|\geq \lambda_{j} \end{array}\right.$$

where $\lambda_{j}$ is the threshold of the j level under the wavelet decomposition, β and m are adjustable parameters, β can be an integer greater than 2, m can be a continuous real number greater than 1, j is the number of wavelet decomposition levels, and k denotes the kth decomposition coefficient at that particular level.

By manipulating the parameter, β, the value of the threshold function at $d_{j,k}=\pm \lambda_{j}$ can be finetuned. When β=2, the value at the connection point of the piecewise function is $\pm 3\lambda_{j}/4$. As β increases, the value at the piecewise point becomes closer and closer to $\pm \lambda_{j}/2$. In this study, a value of β=10 was chosen. 

**Figure 5:**
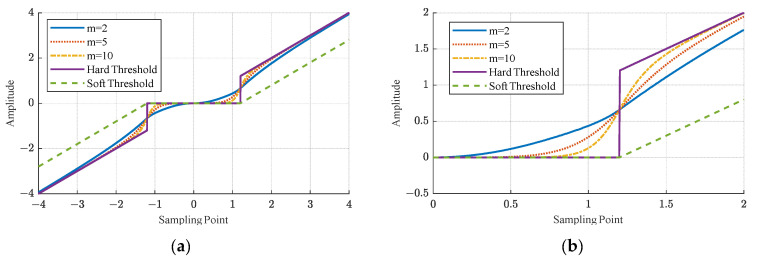
(**a**) The results of applying different threshold functions to a straight line; (**b**) A detailed zoomed-in view of (**a**).

By adjusting the parameter, m, the threshold function can be selected at various decomposition levels. Following the wavelet decomposition, the proportion coefficient between the white noise signal and the energy of the measured signal at the *j*th decomposition level is calculated. A very small proportion coefficient indicates a significant reduction in the noise component of the level; therefore, the partial soft threshold function is utilized for denoising. A significantly larger proportion coefficient suggests that the high-frequency components in the level are predominantly noise; thus, the partial hard threshold function is employed for denoising. The specific parameter, m, for adaptively selecting the threshold function can be determined using the following equation:

(5) $$m_{j}=1+10\frac{E_{n_{j}}}{E_{d_{j}}}$$

where $E_{n_{j}}$ and $E_{d_{j}}$, respectively, are the energy of the white noise and the echo signals in level j under the wavelet decomposition. In this operation, they can be directly viewed as the sum of squares of the high frequency coefficients in this level. The processing results of the adjustable threshold function with different m values are shown in Figure 5. The threshold function, which is predicated on the energy ratio of the wavelet coefficients, exhibits continuity and smoothness, thereby ensuring signal continuity and minimizing the loss of significant coefficients.

**Figure 6:**
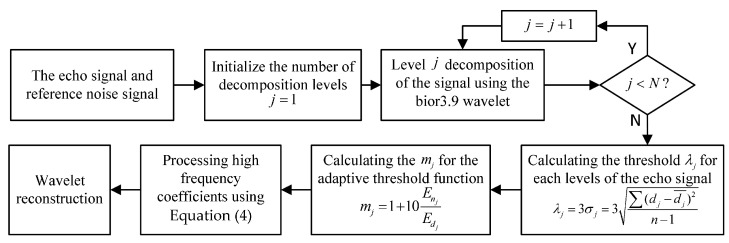
Wavelet denoising algorithm process for the ultrasonic echo signals.

This study used the bior3.9 wavelet to decompose the echo signal. Figure 6 shows the detailed steps of wavelet threshold denoising.

#### 3.1.2. Simulation and Analysis of Wavelet Adaptive Threshold Denoising

**Figure 7:**
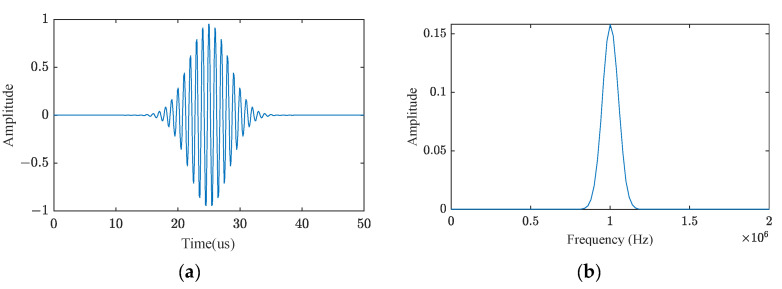
(**a**) Plot of the echo signal based on the Gaussian empirical model; (**b**) Spectral and amplitude analysis of the echo signal.

A simulation experiment was conducted to assess the effectiveness of the wavelet threshold denoising approach proposed in this study for the echo signal noise reduction. This study employs the Gaussian classic model, corresponding to the ultrasonic pulse, to construct an echo signal with a center frequency of 1 MHz, a sampling frequency of 10 MHz, a bandwidth of 200 kHz, and a length of 500, as illustrated in Figure 7.

This study assesses the efficacy of the denoising process, utilizing two primary metrics: the Signal-to-Noise Ratio (SNR) and the Root Mean Square Error (RMSE). The formulas for these calculations are delineated below:

(6) $$SNR=10lg\frac{\sum {f(n)}^{2}}{\sum {\left(f\left(n\right)-{f(n)}^{\prime }\right)}^{2}}$$

(7) $$RMSE=\sqrt{\frac{\sum {\left(f\left(n\right)-{f(n)}^{\prime }\right)}^{2}}{N}}$$

where n is the length of the signal, ${f(n)}^{\prime }$ is the denoised signal, and f(n) is the original signal.

**Figure 8:**
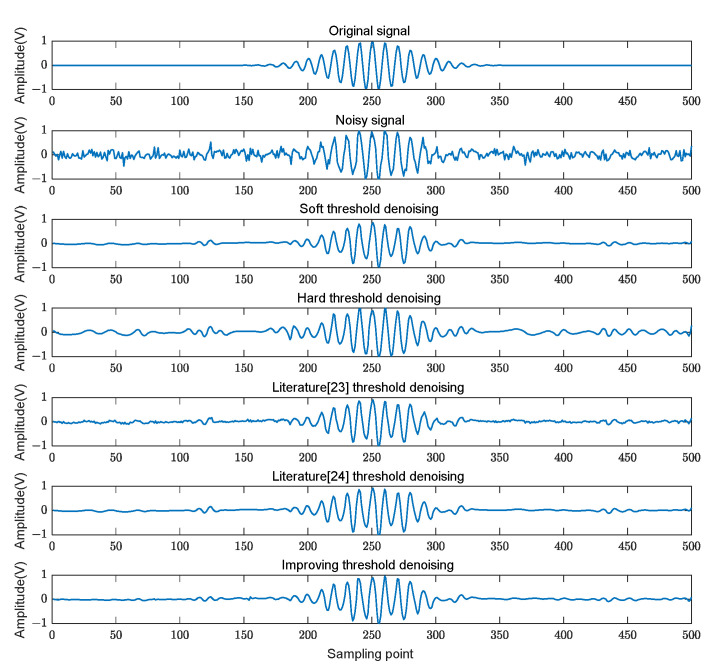
Wavelet denoising effect with different threshold functions.

After the introduction of 5 dB white noise into the ultrasonic echo signal, the denoising procedure was executed utilizing various wavelet thresholding functions. Figure 8 illustrates the comparative denoising efficacy of these methods.

In Figure 8, we choose the denoising effect of the soft and hard thresholding functions and the other two thresholding functions to compare with the adaptive thresholding function in this study. In Table 1, the SNR and RMSE processed by different threshold functions for the same noisy echo signal are shown. In the simulation experiment focused on Gaussian echo signal denoising, the proposed method outperformed the existing techniques, as demonstrated by a superior SNR and a lower RMSE. The improvement in the SNR indicates that the proposed method is more effective in enhancing the clarity and overall quality of the signal, effectively distinguishing it from the noise. Furthermore, the decrease in RMSE highlights the accuracy of the method in closely approximating the original signal, emphasizing its capability to retain the integrity of the signal while mitigating noise interference. The success of the proposed method is largely attributable to its adaptive thresholding function, which intelligently modulates the threshold in response to the signal-to-noise energy ratio, ensuring an optimal balance between the noise suppression and the signal preservation. This approach allows for a more nuanced and efficient denoising process, tailored to the unique characteristics of the echo signal.

### 3.2. Feature Extraction of Ultrasound Echo Signals

#### 3.2.1. Construction of Cross-Correlation Reference Signals Utilizing Estimated Initial Values

In this study, we employed the cross-correlation method to accurately determine the time delays present in echo signals originating from both the anterior and posterior walls of the bladder. This technique calculates the time delay by examining the correlation between a predefined reference signal and the actual echo signals received. The precision of this technique is significantly influenced by the selection of the reference signal. To optimize this process, the study constructs Gaussian empirical models based on the initial echo signals from the various bladder volumes, providing a tailored reference signal for the cross-correlation analysis. This approach not only enhances the precision of identifying the time delays but also improves the real-time performance of the estimation algorithm.

The model fitting challenge fundamentally entails minimizing the residual discrepancy between the observed and theoretical signals, a process known as the least squares model fitting issue [25]. To estimate the parameter vector, θ, constructing a least squares function aimed at optimizing f(θ) is imperative. The formulation of this function leverages the least squares method, with its explicit expression as follows:

(8) $$f\left(\theta \right)=\sum_{i=1}^{N}{x\left(i\right)-s(\theta^{\left(k\right)})}^{2}$$

where N denotes the number of discrete points, *x* denotes the initially collected echo signal, and $s(\theta^{\left(k\right)})$ signifies the parameter vector at the *k*-th iteration of the Gaussian empirical model. Achieving the minimum value of f(θ) indicates an alignment between the model and the observed signals. This alignment represents the best fit, thereby yielding the optimal parameters. This study employs the Gauss–Newton algorithm to address this optimization challenge.

This study introduces a novel strategy to mitigate the initial value sensitivity in parameter estimation methods and the issue of slow convergence rates. This strategy leverages the energy and envelope data extracted from the wavelet-denoised echo signal as a priori knowledge for the parameter estimation. Consequently, this approach facilitates more precise initial value estimations and accelerates the parameter estimation convergence process.

(1) Correlation between the energy and the signal parameters

Considering a Gaussian echo signal with a zero phase and a normalized amplitude, the spectrum expression is:

(9) $$\left|S\left(f\right)\right|=\frac{1}{2}\sqrt{\frac{\pi }{\alpha }}(e^{\frac{{-\pi^{2}(f-f_{0})}^{2}}{\alpha }}+e^{\frac{{-\pi^{2}(f+f_{0})}^{2}}{\alpha }})$$

where f represents the frequency variable, and the energy spectrum is obtained by the square of Equation (9):

(10) $$P_{ss}\left(f\right)={\left|S\left(f\right)\right|}^{2}=\frac{\pi }{4\alpha }(e^{\frac{{-{2\pi }^{2}\left(f-f_{0}\right)}^{2}}{\alpha }}+e^{\frac{{-{2\pi }^{2}\left(f+f_{0}\right)}^{2}}{\alpha }}+2e^{\frac{-{2\pi }^{2}{f_{0}}^{2}}{\alpha }}e^{\frac{-{2\pi }^{2}f^{2}}{\alpha }})$$

The energy of the Gaussian echo signal is derived by integrating f over the interval (−∞,+∞), as delineated in Equation (10).

(11) $$E=\int_{-\infty }^{+\infty }P_{ss}\left(f\right)df=\frac{\beta^{2}}{2}\sqrt{\frac{\pi }{2\alpha }}(1+e^{\frac{-{2\pi }^{2}{f_{0}}^{2}}{\alpha }})$$

When $f_{0}^{2}\geq 0.2333\alpha$, the energy contribution of the exponential term in Equation (11) becomes negligible, thereby simplifying the energy equation to:

(12) $$E_{s}=\frac{\beta^{2}}{2}\sqrt{\frac{\pi }{2\alpha }}$$

Equation (12) demonstrates that the energy of the echo signal correlates with the β and α parameters of the signal.

(2) Relationship between the envelope and the signal parameters

**Figure 9:**
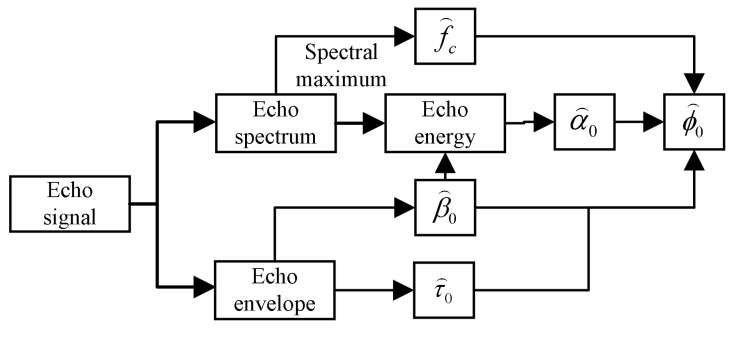
Initial value estimation procedure for model fitting.

The envelope of the ultrasonic echo signal can also obtain the characteristics of the signal for parameter estimation. The envelope of the Gaussian echo signal contains three parameters: β, α, and τ. The maximum value and corresponding time of the envelope signal correspond to Gaussian, respectively. The initial value estimation process of β and τ in the echo model is shown in Figure 9.

Upon determining the initial value of the parameter vector, θ, an objective function is formulated in accordance with Equation (8). This transforms the problem into a nonlinear unconstrained least squares formulation. This study employs the Gauss–Newton algorithm for the solution, recognized for its high computational precision, rapid convergence, minimal computational load, and excellent data fidelity.

**Figure 10:**
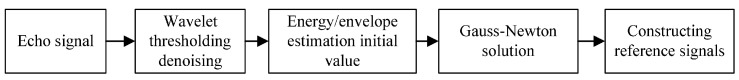
Constructing the reference signal flow.

In this study, the initial echo signal derived from varying bladder volumes served as the parameter subject to the optimization. By employing the Fourier transform and the Hilbert analysis, the initial value information was meticulously extracted, aiming to diminish the number of iterations required during the estimation phase, thereby enhancing both the efficiency and the precision of the parameter estimation. Subsequently, the Gauss–Newton optimization method is applied to ascertain the optimal model parameters, culminating in the reconstruction of the echo signal. The process of constructing the reference signal via the Gauss–Newton algorithm is depicted in Figure 10.

#### 3.2.2. Cross-Correlation Method and Hilbert Envelope Peak Extraction

Following the wavelet denoising of the ultrasonic echo signal and the construction of the reference signal, the objective of this study was to ascertain the time delays associated with the echo signals reflected from both the anterior and posterior walls of the bladder. This was achieved by calculating the correlation between the cleaned echo signals and the reference signal using the cross-correlation method. To accurately identify the time delays of the echoes from the bladder walls, applying the Hilbert Envelope to the results of the cross-correlation function was crucial, enabling the precise extraction of the signal features [26].

The cross-correlation technique calculated the echo arrival time by assessing the similarity between the reference and the measured signals. By using multiple echoes with a certain similarity, the measured signal was interpreted as a superposition of the delayed reference signal and the noise signal. This relationship can be expressed as:

(13) $$S_{e}\left(t\right)=\alpha \times S_{r}\left(t-D\right)+n(t)$$

where α is the attenuation coefficient, D is the propagation time of the echo in the human body, $S_{r}\left(t\right)$ is the reference signal, and n(t) is the noise signal.

The cross-correlation function between the reference signal and the echo signal is given by:

(14) $$R_{re}\left(\tau \right)=E[S_{r}\left(t+\tau \right)S_{e}\left(t\right)]$$

By substituting into the above two equations, we obtain:

(15) $$R_{re}\left(\tau \right)=R_{rr}(\tau -D)$$

In Equation (15), when τ=D, the cross-correlation function attained its maximum value, enabling the estimation of the arrival time of the echo signal from the peak of the cross-correlation function. By applying the Hilbert envelope to extract the peak of the cross-correlation function, the echo distance was determined.

#### 3.2.3. Reference Signal Simulation and Analysis

**Figure 11:**
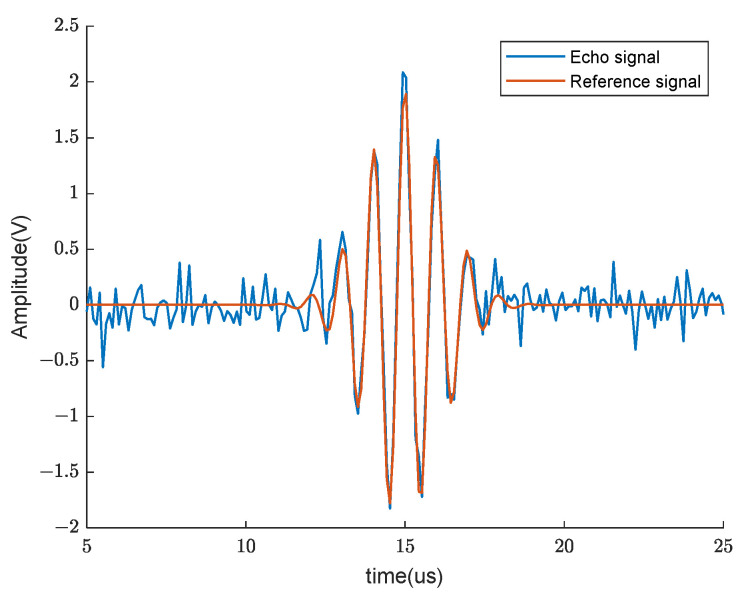
Generate the waveform of the reference signal at an SNR of 5 dB.

To verify the initial value estimation performance of the proposed algorithm, a simulation experiment for constructing the reference signals was conducted. Initially, an echo model was employed to simulate the signals reflected from the anterior and posterior walls of the bladder. The model operated at a sampling frequency of 10 MHz, with parameters $\theta =\left[\beta ,\alpha ,\tau ,f_{0},\phi \right]=[2,\;0.4,\;15,\;1,\;0]$. To simulate real-life noisy bladder echo signals, white noises were added to the signal. The termination criteria for the Gauss–Newton algorithm were set as either a function difference less than $10^{-8}$ or exceeding 500 iterations. For comparison, a fixed initial value of $\theta^{\left(0\right)}=[1\;1\;12\;1\;1]$ was set to contrast with the estimated initial value method, as described in Section 3.2.1. The experiment was repeated 100 times to calculate the average time and the fitting parameter estimates for both algorithms. Figure 11 displays the reference signal fitting under 5 dB white noise, while Table 2 presents the simulation results.

The data clearly demonstrate the superiority of the initial value estimation method over the fixed initial value method in terms of both accuracy and computational efficiency. This is particularly evident under noisy conditions, where the robustness of the initial value estimation method becomes a significant advantage. The drastic increase in the error for the fixed initial value method at a 5 dB noise level, especially in parameters such as β and ϕ, underscores the challenges of working with high noise levels and the importance of selecting an appropriate parameter estimation method.

Moreover, the consistently shorter computation times for the initial value estimation method across all the noise levels suggest that this method is not only more accurate but also more efficient, making it a preferable choice for real-world applications where the time and accuracy are critical.

The comparative analysis between the different noise levels indicates that, as the SNR decreases, the performance gap between the two methods widens, with the initial value estimation method showing a remarkable resilience to noise. This resilience is crucial for applications in environments with high levels of noise, where accurate and efficient parameter estimation is vital.

The experimental data showcase the effectiveness of the initial value estimation method over the fixed initial value method, particularly in noisy conditions. This method not only achieves closer estimates to the true values across various noise levels but also demonstrates a significant advantage in computational efficiency. The resilience of the initial value estimation method to noise highlights its suitability for practical applications where accurate and fast parameter estimation are critical. Given its robust performance, especially at lower signal-to-noise ratios, it emerges as the preferred choice for scenarios demanding high precision and efficiency in parameter estimation amidst noise challenges.

### 3.3. Bladder Volume Fitting

For the bladder volume estimation, the shape of the bladder is commonly modeled as an ellipsoid, triangular prism, cube, or cylinder [27], with the ellipsoid method favored for its accuracy and simplicity [28,29]. This study adopted the assumption that the morphology of the bladder resembles an ellipsoid. By applying the least squares method to fit the ellipsoid shape, we sought to identify the coefficients of the ellipsoid equation that minimize the sum of the squared algebraic distances. The quadratic surface equation f(a,x) is expressed as follows:

(16) $$f\left(a,x\right)=a\cdot x=ax^{2}+by^{2}+cz^{2}+2dxy+2gyz+2hxz+2px+2qy+2rz+I=0$$

In the equation:

$$a={\left(a,b,c,d,g,h,p,q,r,I\right)}^{T},x={\left(x^{2},y^{2},z^{2},xy,xz,yz,x,y,z,1\right)}^{T}$$

The application of the least squares method is designed to identify a specific set of parameters that minimize the sum of the squared distances from the bladder wall position data, as measured by the system, to the ellipsoid surface. Thus, a new loss function is formulated that quantifies the deviation of all data points from the ellipsoid equation. The deviation equation is given by:

(17) $$minG\left(a,b,c,d,g,h,p,q,r,I\right)=\min\left(\sum_{i=1}^{n}e_{i}^{2}\right)=\min\left(\sum_{i=1}^{n}{\left(f\left(x_{i},y_{i},z_{i}\right)-\hat{f}\left(x_{i},y_{i},z_{i}\right)\right)}^{2}\right)$$

where i represents the number of measured data and $e_{i}$ denotes the residual.

In this study, we utilized two linear combinations of the invariant constraints as proposed by Li [30], implementing a numerically stable algorithm that necessitates no additional computational effort while ensuring improved robustness.

## 4. Bladder Volume Estimation Experiment

### 4.1. Experimental Design

**Figure 12:**
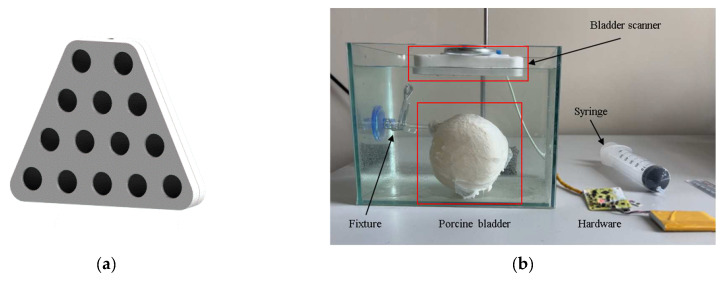
(**a**) Diagram of the bladder scanner structure; (**b**) Experimental scene for measuring porcine bladder urine volume.

The hardware of the bladder fluid volume measuring equipment encompasses a variety of components, such as a circuit system for ultrasonic wave transmission and reception, a microprocessor with its auxiliary circuits, a power supply unit, a signal transmission module, and external connection interfaces. This equipment employs A-type ultrasonic pulse-echo technology for the bladder measurement. As the urine volume accumulates, the bladder undergoes changes in size and shape, potentially expanding in width and height. To secure comprehensive data regarding the positions of the anterior and posterior walls of the bladder, a bladder scanner was designed (refer to Figure 12a). The bladder scanner is composed of 14 ultrasound transducers. During the measurement, the ultrasound transducers were sequentially controlled to emit the ultrasound waves in turn, thereby obtaining the distances between the anterior and posterior walls of the bladder at different positions.

This study conducted a feasibility experiment on the bladder volume estimation algorithm using a porcine bladder obtained from an abattoir, as depicted in Figure 12b. The bladder was submerged in water to closely simulate the ultrasound propagation process in human tissues. The bladder scanner was positioned directly above each model. Before the experiment, the bladder was emptied of air and connected to a silicone tube. During the experiment, water was incrementally added to the porcine bladder in 50 mL steps, culminating in a total of 500 mL to emulate the bladder filling process. Following each injection, the bladder scanner sequentially extracted the echo signals from various locations on the bladder. The algorithm outlined in this study was employed to extract the features of the anterior and posterior walls for the volume estimation.

### 4.2. Experiment Results

**Figure 13:**
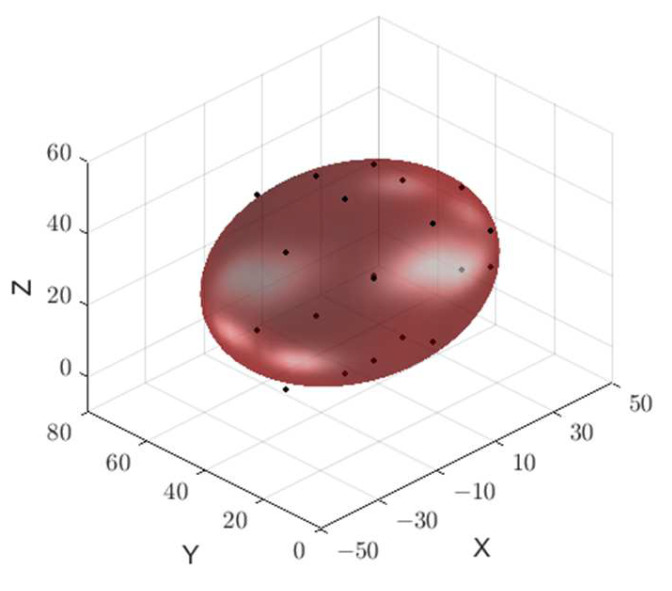
The 150 mL bladder volume fitting results are displayed.

Following the acquisition of distances from both the anterior and posterior bladder walls to the probe, the bladder urine volume was calculated using the algorithm outlined in Section 3. The MATLAB fitting results for a bladder urine volume of 150 mL are depicted in Figure 13, with the experimental measurement yielding a volume of 164 mL.

**Figure 14:**
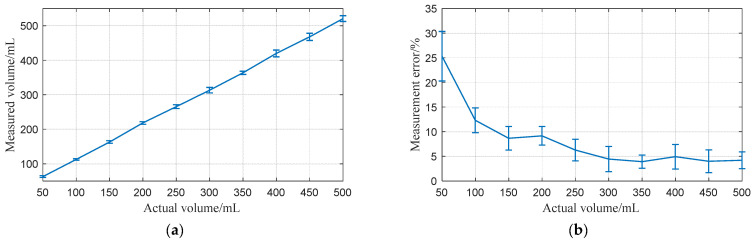
(**a**) Measured versus actual volume for different experiments; (**b**) Measurement error for different experiments. The error bars for three repetitions of each experiment are shown.

Porcine bladders, with volumes ranging from 50 mL to 500 mL, were measured. Initially, the bladders were pre-filled with 50 mL of water, and subsequent injections of 50 mL were administered using a medical syringe. Figure 14 illustrates the comparison between the actual and measured volumes of the porcine bladder across various volume levels.

The high error rates observed in the initial experiments could be attributed to the sensitivity of the algorithm to smaller volumes or limitations in the experimental setup. This suggests potential areas for algorithm refinement or adjustment in the measurement technique to enhance low-volume accuracy.

From a broader perspective, the error decreases as the volume increases, indicating that the algorithm becomes more accurate and reliable with the enlargement of the bladder capacity. This trend may be attributed to the imperfect elliptical shape of the bladder at lower volumes. As the bladder volume increases and becomes more distended, the measurement error correspondingly diminishes, suggesting an improved algorithmic performance in estimating bladder volume under conditions of increased fullness. 

Upon calculation, the average measurement error was determined to be approximately 8.3%, which can provide effective information for the assessment of the bladder volume. This level of accuracy demonstrates the potential applicability of the proposed method in clinical settings, where reliable volume estimation is critical for diagnostic and treatment purposes.

In summary, the experimental data demonstrate the potential of the proposed algorithm for accurate bladder volume estimation, with a notable improvement in accuracy as the volume increases. While the high error rates at lower volumes suggest areas for further refinement, the overall trend indicates the viability of the algorithm for clinical applications, especially in scenarios involving the monitoring of larger bladder volumes. Future studies could focus on refining the algorithm to further minimize the error rate, thereby enhancing the utility and reliability of bladder volume measurements in medical practice.

## 5. Conclusions

To address the issue of low accuracy in portable bladder urine volume measurement devices, this study developed an A-mode ultrasound-based bladder volume estimation algorithm. The algorithm aims to meet the requirements of daily real-time monitoring and improve the portability and accuracy of estimation. A wavelet adaptive denoising method based on energy ratio has been introduced to effectively enhance the SNR of the ultrasound signal and retain the key signal details. Additionally, by conducting a cross-correlation analysis and extracting features from the Hilbert envelope, the algorithm can precisely determine the position of the anterior and posterior walls of the bladder. This information is then used to fit an ellipsoid using the least squares method, resulting in an accurate estimation of the bladder volume. In a simulation experiment of the porcine bladder, the algorithm demonstrated good estimation accuracy, with an average deviation of 8.3%, proving its potential for clinical application in bladder function assessment and related disease diagnosis and care. This study provides an effective algorithm for accurately measuring bladder volume and offers a new technical reference and support for the diagnosis and treatment of bladder-related diseases in the future.

## Figures and Tables

**Table 1:** SNR and RMSE of echo signal after denoising.

Denoising Algorithm	Soft Threshold Denoising	Hard Threshold Denoising	Literature [23] Threshold Denoising	Literature [24] Threshold Denoising	Threshold Denoising in This Study
SNR	9.2778	8.8603	10.0531	10.1305	10.6091
RMSE	0.0813	0.0853	0.0743	0.0737	0.0697

**Table 2:** Mean of the parameter estimation results.

	Method	β	$\alpha \;{\left((MHz\right)}^{2})$	τ (us)	$f_{c}\;(MHz)$	ϕ (rad)	Time (s)
Truth Value	−	2	0.4	15	1	0	−
Noiseless	Fixed initial value method	2.0000	0.4000	10.0000	1.0000	0.0000	0.0519
	Initial value estimation method	2.0000	0.4000	10.0000	1.0000	0.0000	0.0052
20 db	Fixed initial value method	1.9887	0.3916	14.9967	1.0019	−0.0161	0.0653
	Initial value estimation method	1.9887	0.3916	14.9967	1.0019	12.5503	0.0059
10 db	Fixed initial value method	1.9654	0.3739	14.9891	1.0060	12.5116	0.0660
	Initial value estimation method	1.9654	0.3739	14.9891	1.0060	−0.0548	0.0062
5 db	Fixed initial value method	0.9967	0.1145	13.8449	1.0280	5.1260	0.0906
	Initial value estimation method	1.9405	0.3543	14.9795	1.0105	−0.1060	0.0058

## Data Availability

Data are contained within the article.
